# A response to the letter to the editor by Driscoll et al.

**DOI:** 10.1186/s12989-020-00364-0

**Published:** 2020-07-16

**Authors:** Anne T. Saber, Sarah S. Poulsen, Niels Hadrup, Nicklas R. Jacobsen, Ulla Vogel

**Affiliations:** National Research Centre for the Working Environment, Copenhagen, Denmark

**Keywords:** Chronic inhalation study, Cancer, Particle inhalation, Risk assessment, Nanoparticle, Nanomaterial

## Abstract

In response to the Letter to the Editor by Kevin Driscoll et al., we certainly agree that particle clearance halftimes are increased with increasing lung burden in rats, hamsters and mice, whereas complete inhibition of particle clearance has only been observed in rats, and only at high particle concentrations (50 mg/m^3^). Where we disagree with Kevin Driscoll and colleagues, is on the implications of the increased clearance halftimes observed at higher lung burden. We argue that it does not hamper the extrapolations from relatively high dose levels to lower dose levels.

Furthermore, we highlight, again, the challenges of detecting particle-induced lung cancer in epidemiological studies where occupational, particle-induced lung cancer has to be detected on top of the background lung cancer incidence. Almost all available epidemiological studies on carbon black and titanium dioxide suffer from a number of limitations, including lack of control for smoking, the use of background population cancer rates as reference in the US studies, lack of information regarding particle size of the exposure, and incomplete follow-up for cause of death of the study population.

## Main text

First, we would like to thank Kevin Driscoll and colleagues for their interest and for their comments to our Commentary regarding the use of chronic inhalation studies in rats as risk assessment tool [1].

## Response to points 1 &2

Even though we tried our best to be very clear in the commentary, we realize that our statements can be read in different ways. We certainly agree that clearance halftimes are increased with increasing lung burden in mice and rats as we also stated (‘Particle clearance rates in mice, hamsters and rats depend on the lung burden: lower clearance rates are observed with increasing lung burden’). Moreover, complete inhibition of particle clearance has only been observed in rats, and only at high particle concentrations (50 mg/m^3^) [2].

Following inhalation and deposition, particles are cleared away and the clearance is assumed to be exponential regardless of the lung burden, implying that the actual clearance in terms of ‘mass removed per time unit’ will vary tremendously, and the clearance by mass increases with increasing lung burden.

In the study described by Heinrich et al. [3], clearance half-time of a pulmonary tracer (^59^Fe) in the control group was 61–96 days as assessed at different time points during the two-year inhalation study. For rats exposed to 0.8 mg/m^3^ diesel exhaust, the clearance halftime was 94 days after 3 months exposure, and the lung burden was 0.6 mg and thus, 0.3 mg diesel exhaust particles were cleared away from the lungs during 94 days. For rats inhaling 10 mg/m^3^ carbon black nanoparticles, the lung burden at 18 months was 50 mg, and the clearance halftime 363 days, implying that 25 mg (83-fold more material by mass) was removed during 363 days (3.9 fold longer time).

Where we disagree, is on the implications of the increased clearance halftimes observed at higher lung burden. We argue that it does not hamper the extrapolations from relatively high dose levels to lower dose levels. Of note, the higher dose levels in question are at the level of current occupational exposure levels (OELs). Rats get lung cancer at 10 mg/m^3^ TiO_2_ NPs, corresponding to the current Danish OEL of 6 mg/m^3^ Ti, and at 2.5 mg/m^3^ CB, below the current Danish OEL of 3.5 mg/m^3^ CB. In our view, there is evidence that especially CB is mutagenic [4], and CB-induced genotoxicity has been observed at low dose levels in liver [5] and in lungs [6, 7] in mice. The proposed mechanism of action (particle-induced ROS) is primary genotoxicity.

## Response to points 3 and 4

Concerning the comments regarding epidemiological studies, we disagree. We would, again, highlight the challenges of detecting particle-induced lung cancer in epidemiological studies. Occupational, particle-induced lung cancer has to be detected on top of the background lung cancer incidence. The lung cancer incidence is currently ca. 5% in many European countries and has historically been higher, especially in the 1940s to 1970s, i.e. covering the typical calendar period of the epidemiological studies. This means that even if 1% of the exposed persons die from particle-related lung cancer, the resulting odds ratio would at best only be 1.2, an odds ratio that requires large sample sizes to obtain sufficient statistical power. In the commentary, we highlighted a number of issues hampering the detection of particle-induced lung cancer, and that it would require large epidemiological studies with very long follow-up periods and excellent confounder control to detect particle-induced lung cancer on top of smoking-induced lung cancer. This is because there seems to be a long lag time for particle-induced lung cancer (from the coal-miner study) and because particle exposure induce other diseases [8]. In addition, many studies use cancer incidence rates in the background population as reference, which is problematic in some countries like the US. Instead, unexposed workers form the same or similar factories can be used as reference group. Furthermore, none of the studies provided information about physico-chemical properties (including particle-size) of the particles in the pigment exposures. As for the comments regarding mixed occupational exposures, we fully agree that the mixed occupational exposures are problematic and that this makes it difficult to discern the effects of different exposures. In the coal-miner study in question [8], however, the risk estimates for coal dust and silica were mutually adjusted, and thus, the contribution from silica was taken into account.

The cited meta-analysis on TiO_2_-realated epidemiological studies [9] suffers from a number of the highlighted limitations, including lack of control for smoking, the use of background population cancer rates as reference in the US studies, lack of information regarding particle size of the TiO_2_ exposure and that cause of death was only obtained for 19% of the study group. The latter would suggest that a number of the included studies did not have sufficiently long follow-up, thus potentially failing to detect particle-induced lung cancer with long lag times. The lack of information on particle size is potentially very important, since particle size is a major determinant of lung cancer potency of TiO_2_ in rats. P25 TiO_2_ NPs induced lung cancer at 10 mg/m^3^, whereas fine TiO_2_ required 25 times higher air concentrations, inducing lung cancer at 250 mg/m^3^, but not at several lower dose levels.

## Note from the editor

It is clear that there are different views on how to assess and interpret adverse effects seen in experimental animals at impaired particle clearance conditions (also referred to as ‘particle overload’) in light of human health risk assessment. We will close the discussion now in Particle and Fibre Toxicology and will only in the near future consider new scientific findings based on original research.

## Data Availability

Not applicable.
